# Lesion stage-dependent causes for impaired remyelination in MS

**DOI:** 10.1007/s00401-020-02189-9

**Published:** 2020-07-24

**Authors:** Katharina Heß, Laura Starost, Nicholas W. Kieran, Christian Thomas, Maria C. J. Vincenten, Jack Antel, Gianvito Martino, Inge Huitinga, Luke Healy, Tanja Kuhlmann

**Affiliations:** 1 Institut für Neuropathologie, Universitätsklinikum Münster, Pottkamp 2, 48149 Münster, Germany; 2 Max Planck Institut für Molekulare Biomedizin, 48149 Münster, Germany; 3 Montreal Neurological Institute, McGill University, Montreal, Canada; 4 Neuroimmunology Unit, Division of Neuroscience, Institute of Experimental Neurology, IRCCS San Raffaele Hospital, 20132 Milan, Italy; 5 Vita Salute San Raffaele University, 20132 Milan, Italy; 6 Department of Neuroimmunology, The Netherlands Institute for Neuroscience, Amsterdam, The Netherlands

**Keywords:** Multiple sclerosis, Remyelination, Oligodendrocytes, Microglia

## Abstract

Multiple sclerosis (MS) is the most frequent demyelinating disease and a leading cause for disability in young adults. Despite significant advances in immunotherapies in recent years, disease progression still cannot be prevented. Remyelination, meaning the formation of new myelin sheaths after a demyelinating event, can fail in MS lesions. Impaired differentiation of progenitor cells into myelinating oligodendrocytes may contribute to remyelination failure and, therefore, the development of pharmacological approaches which promote oligodendroglial differentiation and by that remyelination, represents a promising new treatment approach. However, this generally accepted concept has been challenged recently. To further understand mechanisms contributing to remyelination failure in MS, we combined detailed histological analyses assessing oligodendroglial cell numbers, presence of remyelination as well as the inflammatory environment in different MS lesion types in white matter with in vitro experiments using induced-pluripotent stem cell (iPSC)-derived oligodendrocytes (hiOL) and supernatants from polarized human microglia. Our findings suggest that there are multiple reasons for remyelination failure in MS which are dependent on lesion stage. These include lack of myelin sheath formation despite the presence of mature oligodendrocytes in a subset of active lesions as well as oligodendroglial loss and a hostile tissue environment in mixed active/inactive lesions. Therefore, we conclude that better in vivo and in vitro models which mimic the pathological hallmarks of the different MS lesion types are required for the successful development of remyelination promoting drugs.

**Electronic supplementary material:**

The online version of this article (10.1007/s00401-020-02189-9) contains supplementary material, which is available to authorized users.

## Introduction

Multiple sclerosis (MS) is the most frequent inflammatory and demyelinating disease of the CNS; it affects approximately 2.3 million people worldwide [15]. Approximately 50% of the patients require a walking aid after 10–15 years of disease duration. The socioeconomic costs are significant; in 2013, the annual costs for MS in the US have been estimated to be approximately 10 billion $ per year [1]. Histopathologically, MS is characterized by multifocal demyelinating lesions, inflammatory infiltrates (macrophages, T cells, and B cells), damaged and reduced numbers of axons, and loss of oligodendrocytes [45]. Based on density and distribution of blood-derived monocytes and CNS-resident microglia (subsequently summarized as myeloid cells) active, mixed active/inactive and inactive lesions can be distinguished as described in an updated histological classification of MS [38].

Demyelinated axons are either remyelinated or remain chronically demyelinated making them especially vulnerable to injury mediated by the immune system or lack of trophic support [25]. Axonal injury and loss are already present in early MS lesion stages and are the underlying cause for disease progression [37, 63, 65]. The formation of new myelin sheaths around axons after a demyelinating event, termed remyelination, represents an endogenous repair process which restores the conduction of action potentials, provides trophic support to axons, and protects against axonal damage [18, 33, 47]. Also, recent histological and imaging studies suggest that remyelination contributes to clinical recovery [5, 46]. In different lesions from the same patient, extent of remyelination can vary significantly [56, 57] and lesion localization may influence the extent of remyelination [2, 29, 57].

In animal experiments, proliferation and migration of oligodendrocyte precursor cells (OPC) as well as their differentiation into mature myelinating oligodendrocytes is required for successful remyelination. These complex processes are regulated by the interaction of OPC and oligodendrocytes with neurons and axons, astrocytes as well as immune cells, such as macrophages/microglia, T cells, and B cells (for review see [24, 25, 27, 42]). In progressive MS, OPC are still present in MS lesions albeit in reduced numbers and unevenly distributed, whereas mature oligodendrocytes are almost completely lacking [11, 39, 69]. These findings resulted in the concept of impaired oligodendroglial differentiation as contributing factor for limited remyelination in chronic MS [25, 62]. In animal studies, several signalling cascades have been identified, inhibiting the differentiation of OPC into mature myelinating oligodendrocytes which may be activated by inflammatory cells present in MS lesions. In contrast, myeloid cells polarized into an M2 (anti-inflammatory) phenotype promote oligodendroglial differentiation in experimental animal studies [52]. Extensive research and drug development efforts have been undertaken to identify drugs which promote oligodendroglial differentiation and by that remyelination [16, 50, 51, 53]. Among the identified drugs were anti-Lingo-1 antibodies and clemastine which were tested in clinical phase II trials [8, 9, 30]. Both compounds successfully promoted remyelination in several demyelinating animal models; however, they had a very modest effect in clinical phase II trials [8, 9, 30].

The view that differentiation of OPC into mature myelinating oligodendrocytes is required for successful remyelination in humans has been challenged recently. Measuring the integration of ^14^C derived from nuclear testing into DNA of oligodendroglial lineage cells Yeung et al. suggest that pre-existing oligodendrocytes and not proliferating OPC may contribute to remyelination in MS [71]. Moreover, Jäkel et al. performed scRNA-seq analysis and identified different subpopulations of OPC and oligodendrocytes in brain tissue samples from MS patients and healthy individuals which only partly overlapped with oligodendroglial subsets identified in mouse brains [34, 48]. They propose that the loss of certain subpopulations and the skewing of the differentiation program to other subclasses of mature oligodendrocytes contribute to impaired remyelination in MS [34].

Here, we demonstrate that in active/demyelinating white matter lesions, mature oligodendrocytes are preserved and that a subset of these lesions displays marked remyelination. In mixed and inactive lesions, oligodendroglial loss is most pronounced in the lesion center suggesting that extended time periods of demyelination contribute to oligodendroglial cell death. Mixed lesions are almost completely lacking remyelination and this lack of remyelination is associated with a relative increase in TMEM119^+^ microglia and iNOS^+^ myeloid cells. Moreover, in vitro experiments demonstrate that supernatants from M1 (pro-inflammatory) polarized primary human microglia, but not M2 or M0 (unstimulated) polarized microglia impair the terminal differentiation of hiOL into myelin basic protein (MBP) positive mature oligodendrocytes.

In summary, our data suggest that there are multiple reasons for remyelination failure in MS depending on lesion stage. Our findings indicate that in active/demyelinating lesions, impaired myelin sheath formation despite the presence of mature oligodendrocytes contributes to remyelination failure, whereas in mixed lesions, loss of oligodendrocytes and a hostile tissue environment prevent successful remyelination. Therefore, for the development of remyelination promoting drugs, new animal models are required which better mimic the different MS lesion stages. Furthermore, drug development efforts promoting remyelination should not only target oligodendroglial differentiation but also other important steps required for successful remyelination, such as proliferation and migration of OPC, myelin sheath formation by mature oligodendrocytes, prevention of oligodendroglial loss as well as modulation of the inflammatory environment.

## Materials and methods

### Materials

The study was performed retrospectively on a collection of paraffin-embedded brain biopsy and autopsy tissue specimens from 62 patients. The cohort comprised 38 biopsy tissue samples from 32 patients and 113 MS lesions (81 tissue blocks) from autopsies from 30 patients. Among the autopsies, 53 tissue blocks with 71 lesions from 17 patients were derived from the autopsy collection of the Institute of Neuropathology, University Hospital Münster. From The Netherlands Brain Bank, Netherlands Institute for Neuroscience, Amsterdam (open access: https://www.brainbank.nl), 28 tissue blocks with 42 lesions from 13 patients were obtained; all material has been collected from donors for or from whom a written informed consent for a brain autopsy and the use of the material and clinical information for research purposes had been obtained by the NBB. Brain biopsies were performed as part of diagnostic evaluation of unclear monofocal lesions that showed atypical MRI findings. None of the study authors were involved in decision-making with respect to biopsy or autopsy. The study was approved by the Ethics Committee of the University of Münster and McGill University (Az: 2016-026-f-S, 2016-165-f-S, 2012-407-f-S, 2011-023-f-S, ANTJ 1988-3). Clinical details are provided in Table 1.

**Table 1:** Clinical details

Total number of patients	62
Biopsies	
Total number of tissue samples	38
Male:female patients	9:23
Median age ± SD	49 ± 14.49 years
Lesion type: activity	
Active/demyelinating	36 94.73% of biopsies
Active/post-demyelinating	2 5.26% of biopsies
Median number of tissue samples analyzed for each individual ± SD (1 tissue sample: 29 patients; 2 tissue samples: 2 patients and 5 tissue samples: 1 patient)	1 ± 0.7
Autopsies	
Total number of lesions	113
Male:female patients	12:18
Median age ± SD	59 ± 14.28 years
Median disease duration ± SD (disease duration unknown for 12 patients)	24 ± 13.58 years
Median postmortem delay ± SD (postmortem delay unknown for 17 patients)	8 h 25 min ± 1 h 47 min
Disease course and number of patients	
RRMS	2
SPMS	16
PPMS	2
Unknown	10
Cause of death	
Cardiovascular failure	6
Respiratory failure/pneumonia	9
Sepsis	3
Euthanasia	3
Other	2
Unknown	7
Lesion type: activity	
Active/post-demyelinating	15 13.27% of autopsies
Mixed	35 30.97% of autopsies
Inactive	63 55.75% of autopsies
Median number of lesions analyzed for each individual ± SD (1 lesion: 5 patients; 2 lesions: 7 patients; 3 lesions: 3 patients; 4 lesions: 7 patients; 5 lesions: 3 patients; 6 lesions: 3 patients; 10 lesions: 1 patient; 14 lesions: 1 patient)	3.5 ± 2.74

*NA* not applicable, *RRMS* relapsing remitting MS, *SPMS* secondary progressive MS, *PPMS* primary progressive MS, *NAWM* normal appearing white matter

### Criteria for determination of lesion activity

All lesions included in our study were located within the white matter of the brain and fulfilled the generally accepted histological criteria for the diagnosis of MS [38]. The lesion classification is based on the updated histological classification of MS lesions by Kuhlmann et al. using immunohistochemistry (IHC) for MBP to detect demyelination and CD68 to determine number and distribution of myeloid cells (comprising blood-derived monocytes and CNS-resident microglia) [38]. Active lesions were hypercellular and characterized by a diffuse infiltration of the complete lesion area with numerous CD68^+^ myeloid cells. The density of these cells was higher than in the surrounding periplaque white matter (PPWM) (directly adjacent to the lesions) and normal appearing white matter (NAWM) (further away from the lesions). In biopsy lesions, NAWM and PPWM were summarized as non-demyelinated white matter (NDWM), since biopsy specimens were frequently fragmented and distance between lesion and non-demyelinated white matter was not always unambiguous. Active lesions were further subdivided into active/demyelinating as well as active/post-demyelinating lesions. In active/demyelinating lesions, numerous myeloid cells containing MBP^+^ myelin degradation products in their cytoplasm were observed, whereas myeloid cells in active/post-demyelinating lesions lacked these myelin breakdown products. Mixed active/inactive lesions (formerly called chronic active lesions, including so called smoldering and slowly expanding lesions) were characterized by a hypocellular lesion center and a rim of activated myeloid cells at the border of the lesion, whereas the lesion center was almost completely depleted of myeloid cells. For simplicity, mixed active/inactive lesions will be subsequently termed mixed lesions. Inactive lesions were hypocellular within the whole lesion area with only few myeloid cells present. The density of myeloid cells in inactive lesions was reduced in comparison to that in NDWM.

### Criteria for semiquantitative analysis of remyelination

In biopsies and autopsies, the extent of remyelination was assessed in all lesions using a semiquantitative score. In biopsies, remyelination was identified as thin, irregular formed myelin sheaths utilizing IHC for MBP. Because biopsy samples frequently display only parts of the lesion, we quantified the extent of remyelination using the following categories: 0 = complete absence of remyelination, 1 = individual oligodendrocytes extending remyelinating processes, 2 = patchy remyelination, 3 = remyelination throughout the sampled lesion area [29].

In autopsy cases, regions of remyelination were identified by thin myelin sheaths by IHC for MBP and pale staining in Luxol fast blue (LFB)–periodic acid Schiff (PAS) staining. In the majority of autopsy cases, complete lesions were sampled. Therefore, the lesions were classified depending on the percentage of lesion area that was remyelinated: 0 = no remyelination or presence only at the lesion border making up less than 10% of the whole lesion area, 1 = remyelination was found in more than 10, but less than 50% of the whole lesion area, 2 = more than 50% of the lesion remyelinated, 3 = completely remyelinated lesion.

### Immunohistochemistry

For IHC, tissue specimens were fixed in 4% paraformaldehyde (PFA) and embedded in paraffin. Biopsy and autopsy tissues were cut in 4-µm-thick sections that were stained with hematoxylin and eosin and LFB-PAS. Immunohistochemical stainings were performed using the Dako REAL^TM^ Detection System (#K5001, Dako) and an automated immunostainer (AutostainerLink 48, Dako). IHC was performed using a biotin–streptavidin technique. In short, sections were deparaffinized and intrinsic peroxidase activity was blocked by incubation with 5% H_2_O_2_ in phosphate-buffered saline (PBS) for 5 min afterwards. Primary antibodies were applied as listed in Supplementary Table 1, online resource. IHC was completed using species-specific biotinylated secondary anti-mouse, rat, or rabbit antibodies followed by incubation with streptavidin/peroxidase complex and the reaction product was developed with diaminobenzidine. For myelin stainings, staining quality was evaluated using vessels or grey matter structures as internal controls. Stained sections that did not show a positive staining signal for e.g. NOGOA or OLIG2 in the normal appearing white or grey matter were excluded from further analyses. Therefore, and due to limited tissue material of some biopsies, number of analyzed lesions varies between different quantifications. For quantitative evaluation, sections of interest stained by IHC were analyzed at 100-fold magnification using a morphometric grid. For quantifications of small lesions, the whole lesion area was analyzed and for quantifications of more extensive lesions, at least ten visual fields per region (e.g. lesion center, lesion border, PPWM etc.) were analyzed. For each region of interest, average counts per square millimeter were calculated and compared by statistical analysis. In autopsy lesions, we analyzed different lesion areas. The edge between the lesion itself and NDWM could be identified in all stainings due to different tissue structure in the lesion. Lesion border was defined as the visual fields directly adjacent to the edge inside the lesion at 100-fold magnification. PPWM was defined as the visual fields directly adjacent to the edge outside the lesion at 100-fold magnification. NAWM was characterized as further away from the lesion, at least five visual fields at 100-fold magnification distant from PPWM. As center, we defined the region furthest away from all lesion borders. The region of interest termed “between center and border” was defined as halfway between lesion border and lesion center (see also Fig. 2a). The ratio (eg. ratio of TMEM119^+^/CD68^+^ cells) was determined by staining and quantifying the numbers of positive cells for the individual markers on consecutive sections.

For double IHC, primary antibodies derived from different species were used. Sections were deparaffinized and blocked in 10% PBS/10% fetal calf serum (FCS) for 20 min. Afterwards, tissues were incubated with the primary antibodies overnight at 4 °C. After three washing steps with PBS, Alexa Fluor 488 and Cy3 coupled secondary antibodies (1:250, Jackson ImmunoResearch Laboratories) were applied for 1 h at room temperature. DAPI was added in the second washing steps to counterstain the nuclei using Roti Mount FluorCare DAPI (Dako).

### Generation of induced pluripotent stem cell-derived oligodendrocytes

iPSC were kindly provided by Prof. Gianvito Martino, San Raffaele Hospital Milan (ethic approval from Banca INSpe). hiOL were generated as described previously [22]. In short, 1.5*10^5^ iPSC-derived neural progenitor cells (NPC) were differentiated as described [60] and seeded onto one well of a matrigel (corning)-coated 12-well plate in NPC medium consisting of equal parts of neurobasal (Invitrogen) and DMEM-F12 medium (Invitrogen) with 1:100 B27 supplement lacking vitamin A (Invitrogen), 1:200 N2 supplement (Invitrogen), 1% penicillin/streptomycin/glutamine (PSG), 150 µM ascorbic acid (AA), 3 µM CHIR99021 (Axon Medchem) and 0.5 µM SAG (Cayman Chemical). The next day, cells were lentivirally transduced with a polycistronic lentiviral vector containing the coding regions of human SOX10, OLIG2, and NKX6.2 followed by an IRES-pac cassette allowing puromycin selection for 16 h. The following day, cells were washed and allowed to recover in NPC medium for 1 day. Subsequently, medium was replaced by glial induction medium (GIM) consisting of DMEM-F12 with 1:100 B27 supplement lacking vitamin A, 1:200 N2 supplement, 1% PSG, 1 µM SAG, 10 ng/mL NT3 (Peprotech), 10 ng/mL PDGF-AA (Peprotech), 10 ng/mL IGF-I (Peprotech), 100 µM AA (Sigma), 1:1000 Trace Elements B (Corning), 15 ng/mL T3 (Sigma) and 1 ng/mL bFGF-2 (Peprotech). After 4 days, GDM consisting of DMEM-F12 with 1:100 B27 supplement lacking vitamin A, 1:200 N2 supplement, 1% PSG, 10 ng/mL NT3, 50 µM dbCAMP (Sigma), 10 ng/mL IGF-I, 100 μM AA, 1:1000 Trace Elements B and 60 ng/mL T3 was applied to the cells. Medium was changed every other day. From day 3 to day 8 of differentiation, 0.75 µg/mL puromycin was applied to remove non-transduced cells.

Supernatants of primary microglia and respective media controls were applied from day 4 to day 21 of differentiation to assess early differentiation. To elucidate effects on proliferation, terminal differentiation and cell death untreated hiOL were sorted for O4 at day 21 of differentiation and subsequently treated with supernatants of primary microglia and media controls until day 35 of differentiation.

### Flow cytometry of hiOL

hiOL were sorted and quantified for O4 by utilizing anti-O4-APC antibody according to manufacturer’s instructions (Miltenyi). Briefly, hiOL were detached by accutase. Next, cells were washed, filtered through a 40 µm filter and cell numbers were determined. Subsequently, 2 µL of anti-O4-APC antibody per 1*10^6^ cells were applied and incubated for 10 min at 4 °C in the refrigerator. Afterwards, cells were washed and analyzed and sorted using a FACSAria IIu cell sorter (BD Biosciences). O4^+^ hiOL, identified using unstained cells and isotype controls, were immediately seeded in GDM and used for further experiments analyzing the effects of microglia supernatants. Gating strategy for O4^+^ cells was described previously [67]. Sorted hiOL contain less than 1% GFAP positive cells (data not shown).

### Immunocytochemistry

For immunocytochemistry (ICC), cells were fixed in 4% PFA. After three washing steps, blocking buffer consisting of 5% normal goat serum/5% FCS in PBS was applied for 30 min. Next, anti-O4 antibody (R&D Systems) was applied in a dilution of 1:1000 in blocking buffer and incubated at 4 °C overnight. The next day, cells were washed three times and Alexa Fluor-conjugated secondary antibody was applied for 1 h at RT. After three additional washing steps, 0.1% triton was applied to permeabilize the cells for 10 min followed by additional three washing steps. Next, anti-MBP (abcam), anti-Ki-67 (abcam) or anti-cleaved caspase 3 (R&D Systems) antibodies were applied in blocking buffer and cells were incubated for 1 h at RT. For antibody dilutions see Supplementary Table 1, online resource. After three washing steps, Alexa Fluor-conjugated secondary antibody was applied for 1 h at RT. Cells were again washed three times and subsequently visualized on a Leica DMI6000 B inverted microscope. DAPI was used to counterstain the nuclei.

### Generation of supernatants of primary microglia

Primary human microglia were isolated from fetal and adult tissue samples, followed by standard culturing and activation techniques as previously described [20]. Briefly, adult microglia were derived from surgically resected brain tissue, removed for the treatment of nonmalignant temporal lobe epilepsy. The tissue provided was outside of the suspected focal site of epilepsy pathology, histopathological changes were excluded by an experienced neuropathologist, and histologically healthy specimens were included. Tissue was obtained in pieces < 1 mm^3^ and treated with DNase (Roche) and trypsin (Thermo Fisher) for 30 min at 37 °C. Following dissociation through a nylon mesh (37 μm), the cell suspension was separated on a 30% Percoll gradient (GE Healthcare) at 31,000 × g for 30 min. Glial cells (oligodendrocytes and microglia) were collected from underneath the myelin layer, washed, and plated. Microglia were separated by the differential adhesion properties of the cells and plated in minimum essential medium (MEM; Sigma-Aldrich) supplemented with 5% FBS (Wisent), 0.1% penicillin/streptomycin (Thermo Fisher), and 0.1% l-glutamine (Thermo Fisher). Human fetal microglia were isolated from CNS tissue (17–23 weeks of gestation), obtained from the University of Washington Birth defects research laboratory (BDRL, project#5R24HD000836-51). Briefly, brain tissue was minced and treated with DNase/trypsin. Tissue was then dissociated through a nylon mesh and cells were plated in high glucose DMEM supplemented as above. After 10–14 days in culture, floating microglia were harvested and plated in supplemented DMEM. The purity of the cultures was routinely higher than 90% [21, 41].

For M1 stimulation, cells were treated with human granulocyte–macrophage colony-stimulating factor (GM-CSF, 5 ng/mL, PeproTech) for 5 days followed by 1 h stimulation with IFNγ (20 ng/mL, Invitrogen) and 48 h stimulation with lipopolysaccharide (LPS) (serotype 0127:B8, 100 ng/mL, Sigma-Aldrich). For M2 stimulation, cells were treated with macrophage colony-stimulating factor (M-CSF, 25 ng/mL, PeproTech) for 5 days followed by 48 h stimulation with IL-4 (20 ng/mL, Invitrogen) and IL-13 (20 ng/mL, PeproTech). All supernatants were collected 48 h following activation; base media with addition of cytokines, in the absence of cells were used as controls.

### RNA isolation and qRT-PCR

RNA isolation and qRT-PCR were performed to confirm the polarization of microglia. Briefly, following collection of supernatants, cells were washed once with warm PBS and lysed in TRIzol reagent (Invitrogen). Total RNA extraction was performed using standard protocols followed by DNAse treatment according to the manufacturer’s instructions (Qiagen). For gene expression analysis, random hexaprimers and Moloney murine leukemia virus reverse transcriptase were used to perform standard reverse transcription. Analysis of individual gene expression was conducted using TaqMan probes to assess expression relative to *GAPDH*.

### Statistical analysis

All statistics were calculated using the GraphPad Prism 5 software (GraphPad Software). To compare two groups, Student’s two-tailed *t* test was applied. For comparison of three or more groups, Bonferroni-corrected one-way ANOVA was performed. All tests were classified as significant if the *p* value was less than 0.05 (*p < 0.05, **p < 0.01, ***p < 0.001).

## Results

### Activity and localization of MS lesions

We analyzed 153 lesions from 62 patients. To classify the lesions we used CD68 and MBP as suggested in the updated histological classification for MS [38]. All biopsy tissue samples were classified as active lesions; among them, 36 as active/demyelinating and 2 as active/post-demyelinating. Among the autopsy tissue samples, we found 15 active (all active/post-demyelinating lesions), 35 mixed, and 63 inactive lesions. In patients from which we had four or more lesions available (*n* = 13, all autopsies), we observed predominantly a mixture of different lesion types. Additionally, three patients displayed only inactive and one patient only active lesions. Of the lesions, 26% were located subcortically, 24% periventricularly, and 9% within the cerebellum. The remaining 41% of the lesions were located in the brain hemispheres, but were neither adjacent to the cortex nor the ventricular system.

### Oligodendrocytes are preserved in active/demyelinating lesions, but lost in the center of mixed lesions

**Fig. 1:**
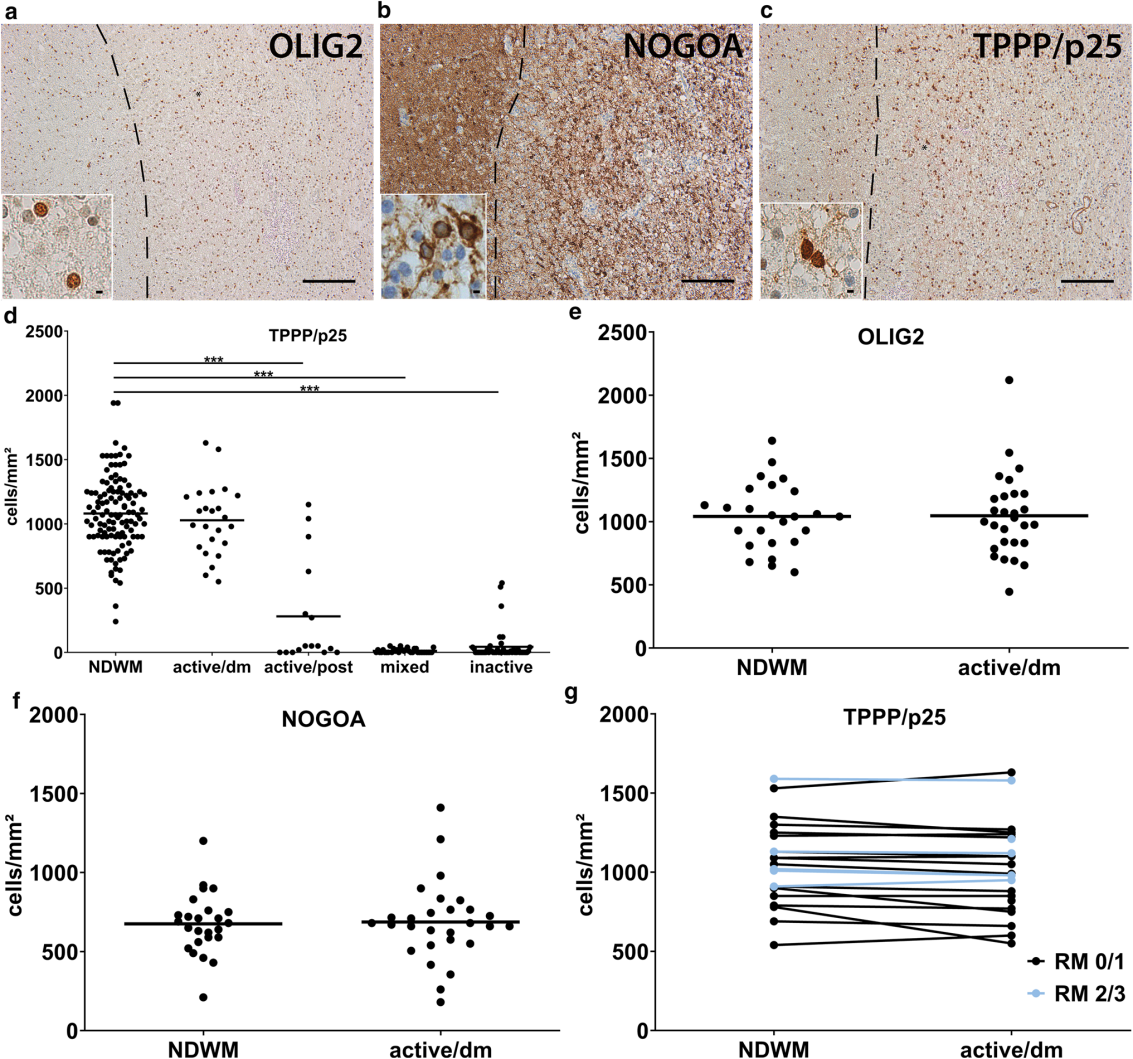
Preservation of oligodendrocytes in active/demyelinating lesions. **a**–**c** OLIG2^+^, NOGOA^+^ and TPPP/p25^+^ oligodendrocytes were identified using IHC. Inserts show oligodendrocytes in higher magnification. **d** Quantification of oligodendrocytes in different lesion types and NDWM using TPPP/p25. **e**, **f** Quantification of OLIG2^+^ and NOGOA^+^ oligodendrocytes demonstrate comparable cell numbers in active/demyelinating as well as in NDWM. **g** When comparing tissue samples containing NDWM and active/demyelinating lesions, no significant differences in numbers of TPPP/p25^+^ oligodendrocytes were observed. Lesions with marked remyelination are indicated in blue, lesions with limited remyelination in black. Scale bars in a to c: 200 µm, scale bars in the inserts in a to c 6.25 µm. *OLIG2* oligodendrocyte transcription factor, *TPPP/p25* tubulin polymerization promoting protein, *NDWM* non-demyelinated white matter, *active/dm* active/demyelinating lesions, *active/post* active/post-demyelinating lesions, *mixed* mixed active/inactive lesions, *inactive* inactive lesions, *RM 0/1* remyelination score 0 or 1, *RM 2/3* remyelination score 2 or 3

We examined the numbers of oligodendrocytes in MS tissue sections using different oligodendroglial markers, such as OLIG2, NOGOA, and tubulin polymerization promoting protein (TPPP/p25) (Fig. 1a–c). OLIG2 labels OPC as well as mature oligodendrocytes, whereas NOGOA and TPPP/p25 are only expressed by mature oligodendrocytes (Fig. 1a, b) [31, 40]. However, our data suggest that NOGOA and TPPP/p25 do not label exactly the same oligodendroglial populations as the absolute numbers of NOGOA^+^ and TPPP/p25^+^ cells differ. In a first step, we quantified the number of TPPP/p25^+^ oligodendroglial lineage cells in NDWM (*n* = 117), active/demyelinating (*n* = 24), active/post-demyelinating (*n* = 16), mixed (*n* = 34) and inactive lesions (*n* = 46). The numbers of TPPP/p25^+^ oligodendrocytes were significantly reduced in active/post-demyelinating, mixed and inactive lesions, but not in active/demyelinating lesions compared to NDWM (Fig. 1d). To validate TPPP/p25 numbers and further characterize oligodendroglial lineage cell numbers in NDWM as well as active/demyelinating lesions, we quantified the numbers of OLIG2^+^ and NOGOA^+^ oligodendrocytes and observed no significant differences (Fig. 1e, f). Similarly, when comparing individual lesions which contained NDWM and active/demyelinating lesion areas, no marked decrease in the numbers of oligodendrocytes was observed either (Fig. 1g; Supplementary Fig. 1, online resource).

**Fig. 2:**
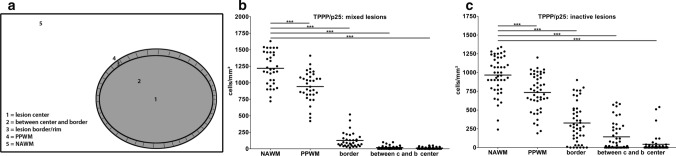
Loss of oligodendrocytes in mixed and inactive lesions. **a** Schematic drawing indicating the different areas in which oligodendroglial cell numbers were quantified. **b**, **c** Immunohistochemistry for TPPP/p25 revealed continuous decrease in TPPP/p25^+^ oligodendrocytes from NAWM to the lesion center in mixed and inactive lesions. *TPPP/p25* tubulin polymerization promoting protein, *PPWM* periplaque white matter, *NAWM* normal appearing white matter, *border* lesion border/rim, *between c and b *between center and border of lesion, *center* lesion center

Next, we analyzed oligodendroglial numbers in mixed lesions from which we had the total lesion area available in more detail. In formalin-fixed paraffin-embedded autopsy material, TPPP/p25 labels oligodendroglial lineage cells more reliably than OLIG2 or NOGOA suggesting that the TPPP/p25 epitope is more stable than NOGOA and OLIG2 epitopes identified by the appropriate antibodies. Therefore, we focused on TPPP/p25 to quantify the numbers of oligodendrocytes in autopsy material. The numbers of TPPP/p25^+^ oligodendrocytes in NAWM, PPWM, at the lesion border, between the lesions border and the center as well as in the lesion center were determined (Fig. 2a). In mixed lesions, the border represents the rim which contains myeloid cells. We observed a significant decrease in oligodendroglial numbers in the different lesion areas including the border as well as PPWM compared to NAWM (Fig. 2b). Similar changes in oligodendroglial numbers were also observed in inactive lesions; however, the oligodendroglial loss was more pronounced in the border of mixed compared to inactive lesions (Fig. 2b, c).

These results demonstrate that there is no major loss of oligodendrocytes in active/demyelinating lesions, but a decrease of oligodendroglial numbers in the lesion center in mixed and inactive lesions.

### Presence of marked remyelination in a subset of active/demyelinating lesions

**Fig. 3:**
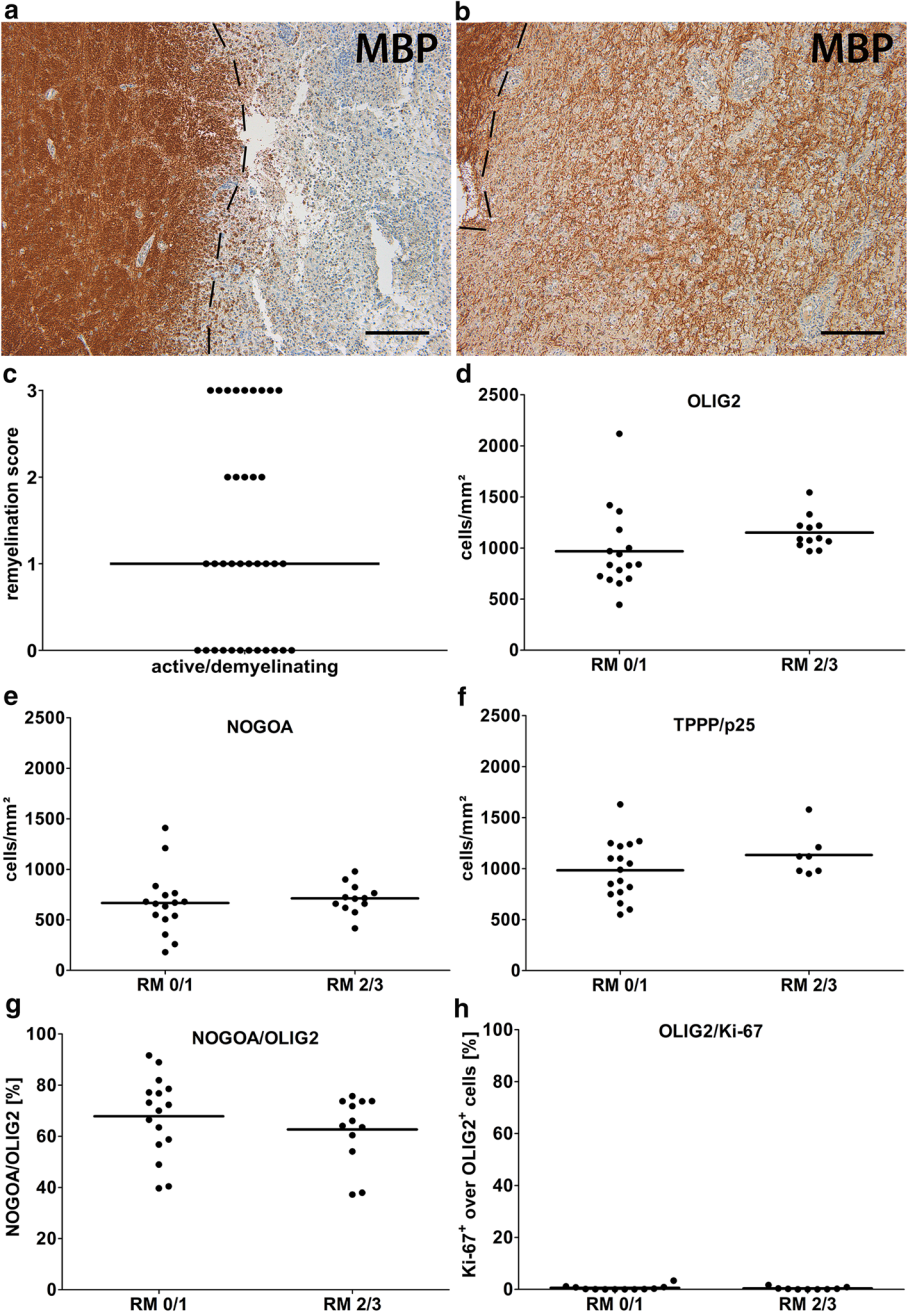
No differences in oligodendroglial cell numbers in active/demyelinating lesions with and without marked remyelination. **a**, **b** A lesion with no remyelination (score 0) is shown in **a**, whereas in **b,** a lesion with marked remyelination (score 3) is displayed (IHC for MBP). **c** In 14 out of 36 (= 39%) of the active/demyelinating lesions, we observed marked remyelination (score 2/3). **d**–**f** Quantification of OLIG2^+^, NOGOA^+^ and TPPP/p25^+^ cells in active/demyelinating lesions with (score 2/3) and without (score 0/1) marked remyelination. **g** No significant difference in the ratio of NOGOA^+^ cells over OLIG2^+^ in lesions with and without marked remyelination. **h** Double IHC for Ki-67 and OLIG2 demonstrated that only few OLIG2^+^ cells express Ki-67**.** Scale bars in **a** and **b**: 200 µm. *MBP* myelin basic protein, *OLIG2* oligodendrocyte transcription factor 2, *TPPP/p25* tubulin polymerization promoting protein, *active/demyelinating* active/demyelinating lesions, *RM 0/1* remyelination score 0 or 1, *RM 2/3* remyelination score 2 or 3

Next, we studied the correlation between oligodendrocytes and remyelination in more detail. Results from longitudinal imaging studies suggest that remyelination occurs within the first 5–6 months after initiation of a demyelinating event [6, 13]. Therefore, we focused on active/demyelinating lesions from biopsies (*n* = 36), since these are the lesions with the highest probability of ongoing remyelination. To quantify the extent of remyelination, we used a semiquantitative score reaching from 0 (no remyelination) to 3 (complete remyelination of the sampled lesion area) (Fig. 3a, b). In active/demyelinating lesions, 14 out of 36 lesions (= 39%) displayed marked remyelination (score 2 or 3) (Fig. 3c). We then compared the numbers of oligodendrocytes in lesions with marked versus limited remyelination (score 0 and 1) (Fig. 3d–f). We observed no significant differences in the numbers of OLIG2^+^, NOGOA^+^ and TPPP/p25^+^ oligodendrocytes in lesions with marked compared to limited remyelination. Additionally, we also compared the ratio of NOGOA^+^/OLIG2^+^ oligodendrocytes which is equivalent to the proportion of mature oligodendrocytes in the total oligodendroglial population. No significant difference between lesions with and without marked remyelination was observed (Fig. 3g). Furthermore, we quantified the percentage of actively dividing oligodendrocytes using double IHC for OLIG2 and Ki-67 (Fig. 3h). We focused our analyses on a subset of active/demyelinating lesions with relatively high numbers of proliferating cells identified by IHC for Ki-67 alone (*n* = 21). The percentage of actively dividing OLIG2^+^/Ki-67^+^ oligodendrocytes was below 2% in all lesions studied and no differences between lesions with and without marked remyelination were detected (Fig. 3h).

In summary, these data suggest that marked remyelination occurs in a subset of active lesions. However, no correlation between oligodendroglial cell numbers, the ratio between mature and total oligodendroglial cell numbers or oligodendroglial proliferation, and the presence of marked remyelination was observed. These results suggest that in lesions with low remyelination scores impaired myelin sheath formation but not reduced oligodendroglial differentiation contributes to lack of remyelination.

### Reduced remyelination in mixed lesions

**Fig. 4:**
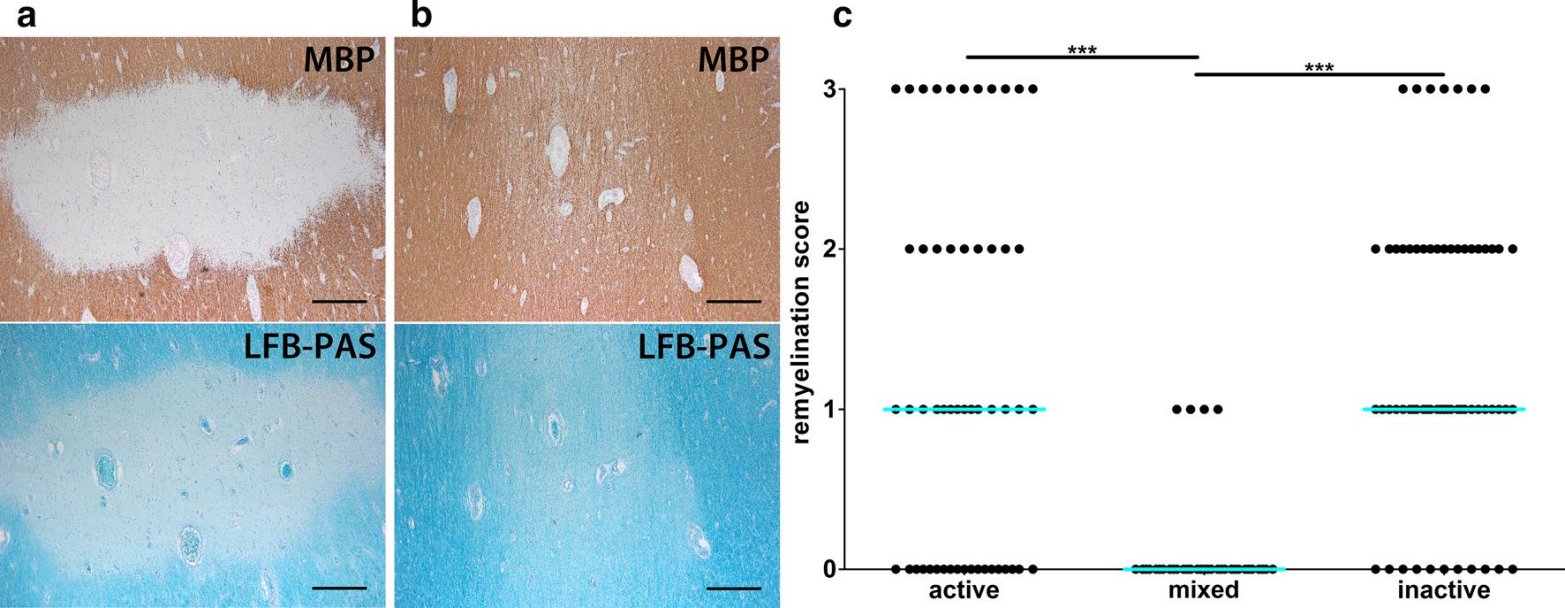
Almost complete lack of remyelination in mixed lesions. **a**, **b** Pictures display an inactive lesion with no remyelination (score 0) (**a**), whereas **b** shows a completely remyelinated shadow plaque (score 3). Upper panels in **a** and **b** show IHC for MBP, lower panels in **a** and **b** display LFB-PAS staining. **c** Semiquantitative analysis of remyelination reveals an almost complete lack of remyelination in mixed lesions (**c**). Scale bars in **a** and **b**: 500 µm. *MBP* myelin basic protein, *LFB-PAS* luxol fast blue–periodic acid Schiff; *active* active lesions, *mixed* mixed active/inactive lesions, *inactive* inactive lesions

In a next step, we compared the extent of remyelination in active (*n* = 53), mixed (*n* = 35) and inactive (*n* = 63) lesions. In active lesions (active/demyelinating as well as active/post-demyelinating) and inactive lesions, a variable extent of remyelination was observed ranging from lesions completely lacking remyelination (score 0) (Fig. 4a) to completely remyelinated lesions (score 3) (Fig. 4b). However, mixed lesions displayed a more uniform pattern with the vast majority of lesions lacking remyelination (31 out of 35 lesions); while the remaining lesions (4 out of 35) displayed remyelination of less than 50% of the lesion area (score 1). The difference in the extent of remyelination between mixed and active as well as inactive lesions was highly significant (*p* < 0.001) (Fig. 4c).

### Higher percentages of TMEM119^+^ and iNOS^+^ myeloid cells are associated with less remyelination in mixed lesions

**Fig. 5:**
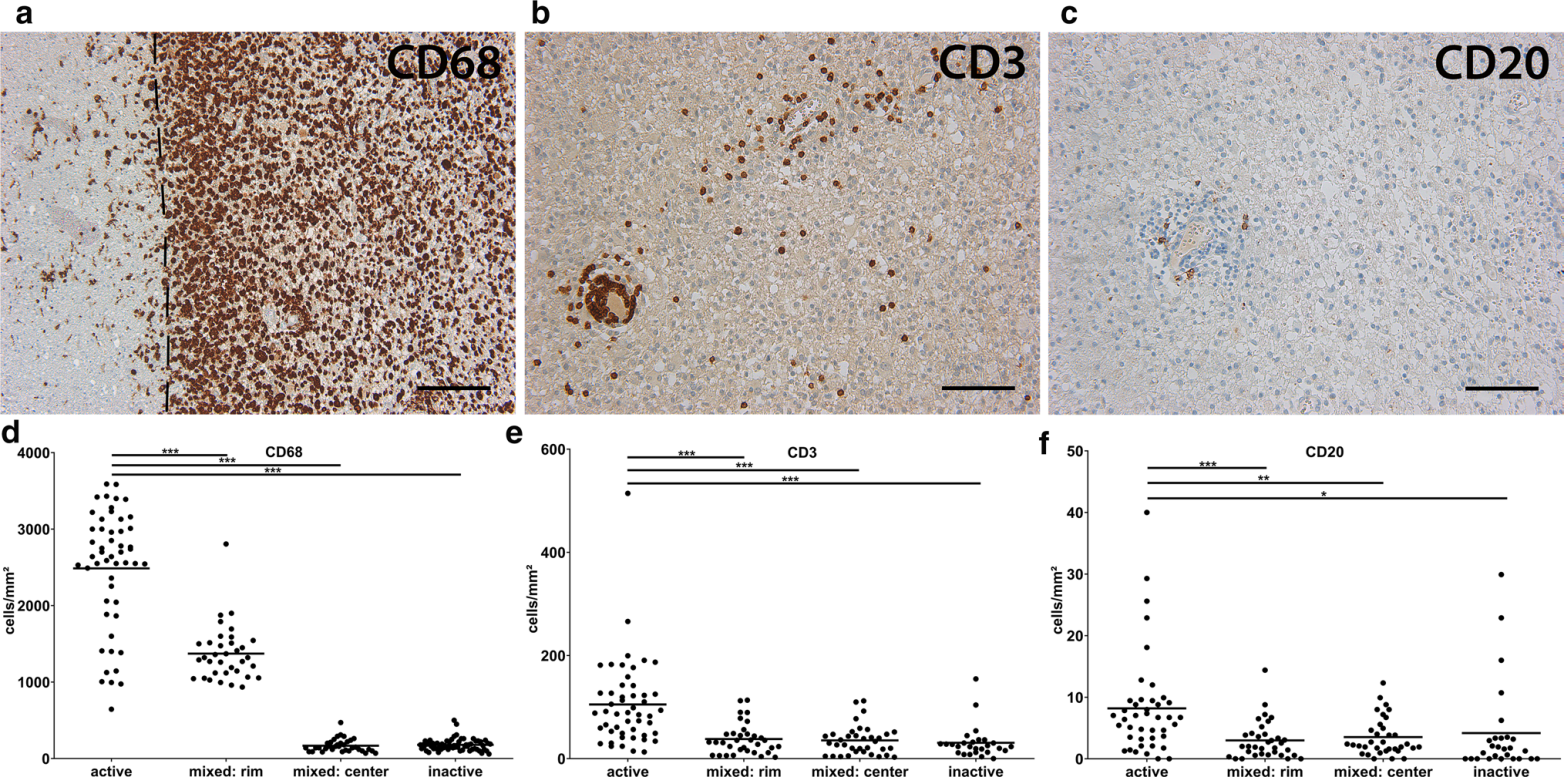
Inflammatory environment in different lesion types. **a**–**c** IHC demonstrates numerous CD68^+^ myeloid cells, fewer CD3^+^ T cells and few CD20^+^ B cells in an active lesion. All pictures are taken from the same active lesion. **d**–**f** Quantification of CD68^+^, CD3^+^ and CD20^+^ cells in different lesion types. Please note the different y-axes. Scale bar in **a**: 200 µm, scale bars in **b** and **c**: 100 µm. *CD3/20/68* cluster of differentiation 3/20/68, *active* active lesions, *mixed: rim*  rim of mixed active/inactive lesions, *mixed: center* center of mixed active/inactive lesions, *inactive* inactive lesions

To investigate whether the inflammatory milieu may influence the outcome of remyelination, we examined the composition of inflammatory infiltrates in active, mixed, and inactive lesions (Fig. 5a–f). Myeloid cells were the dominating inflammatory cell population in all lesion types; however, the numbers of myeloid cells in inactive lesions as well as in the center of mixed lesions were significantly lower compared to active and the rim of mixed lesions (Fig. 5d). Highest numbers of T and B cells were found in active lesions (Fig. 5e, f).

**Fig. 6:**
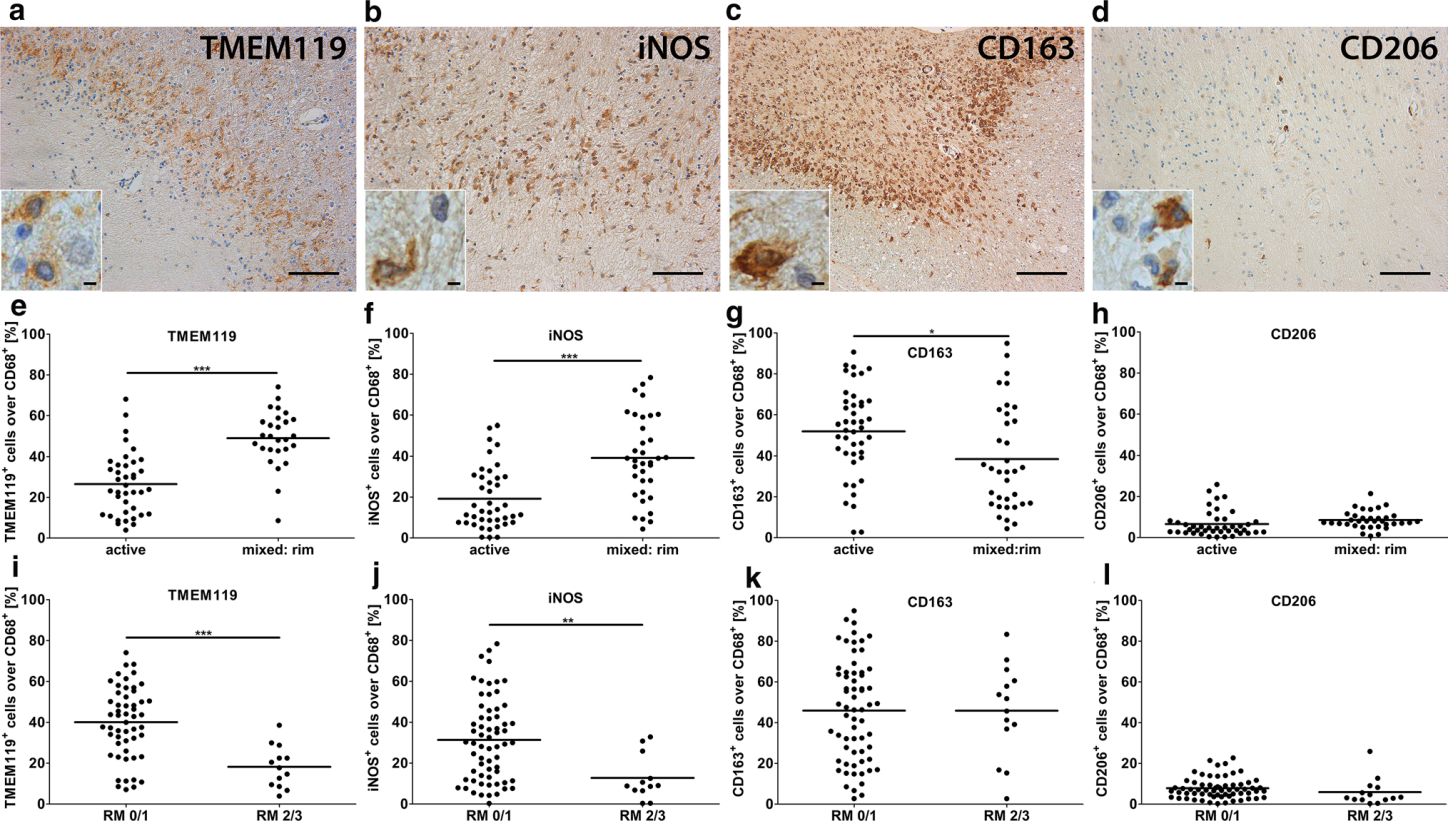
Relative increase in the numbers of TMEM119^+^ and iNOS^+^ myeloid cells in the rim of mixed lesions. **a**–**d** IHC for TMEM119, iNOS, CD163 and CD206 was performed. Inserts show labelled cells in higher magnification. **e**–**g** Quantification demonstrated a significant relative increase in the ratio of TMEM119^+^/CD68^+^ and iNOS^+^/CD68^+^ myeloid cells and a relative decrease of CD163^+^/CD68^+^ myeloid cells in the rim of mixed lesions compared to active lesions. **h** No differences in the number of CD206^+^ over CD68^+^ cells were observed between active lesions and the rim of mixed lesions. **i**, **j** Comparably, we observed a relative increase in the ratio of TMEM119^+^/CD68^+^ and iNOS^+^/CD68^+^ in lesions with limited (score 0/1) compared to lesions with marked remyelination (score 2/3). **k**, **l** No differences were found between lesions with and without marked remyelination with respect to the ratio of CD163^+^/CD68^+^ and CD203^+^/CD68^+^ cells. Scale bars in **a**, **b** and **d**: 100 µm, scale bar in **c**: 200 µm, scale bar in insert in **a**–**d**: 6.25 µm. *TMEM119* transmembrane protein 119, *iNOS* inducible nitric oxide synthase, *CD68/163/206* cluster of differentiation 68/163/206, *active* active lesions, *mixed: rim* rim of mixed active/inactive lesions, *RM 0/1* remyelination score 0 or 1, *RM 2/3* remyelination score 2 or 3

Due to the dominance of the myeloid cell population and based on a plethora of literature describing either detrimental or beneficial effects of myeloid cells on remyelination, we hypothesized that those myeloid cells may determine the outcome of remyelination. We focused our subsequent analyses on active lesions and the rim of mixed lesions, since the center of mixed lesions as well as inactive lesions was almost completely depleted of myeloid cells. To characterize the myeloid cell population in more detail, we stained for TMEM119, a marker of homeostatic microglia, iNOS, a pro-inflammatory marker as well as CD163 and CD206, two markers associated with an anti-inflammatory phenotype of myeloid cells (Fig. 6a–d). The highest absolute number of myeloid cells was identified by CD163 (mean ± SEM: 906 ± 70) followed by TMEM119 (mean ± SEM: 640 ± 36) and iNOS (mean ± SEM: 468 ± 38), whereas fewer myeloid cells expressed CD206 (mean + SEM: 128 ± 12) (Supplementary Fig. 2a–e, online resource). When analyzing the ratio of TMEM119^+^/CD68^+^, iNOS^+^/CD68^+^ and CD163^+^/CD68^+^ cells, we saw a significant increase in the percentage of TMEM119^+^ and iNOS^+^ over CD68^+^ myeloid cells in the rim of mixed lesions compared to active lesions (Fig. 6e, f). The percentage of CD163^+^ cells was significantly reduced in the rim of mixed lesions, whereas no difference in the number of CD206^+^ myeloid cells was observed (Fig. 6g, h). Furthermore, when comparing the ratio of TMEM119^+^, iNOS^+^, CD163^+^ and CD206^+^ over CD68^+^ cells in active and mixed lesions with limited (score 0/1) or marked remyelination (score 2/3), we observed a relative increase in TMEM119^+^ and iNOS^+^ myeloid cells in lesions with limited remyelination.

In summary, these data demonstrate a relative increase of TMEM119^+^ homeostatic microglia and iNOS^+^ pro-inflammatory myeloid cells in the rim of mixed lesions. Furthermore, a relative increase in TMEM119^+^ and iNOS^+^ myeloid cells is associated with reduced remyelination in active and mixed lesions.

### Supernatants of M1, but not M0 or M2 polarized microglia inhibit terminal differentiation of hiOL

**Fig. 7:**
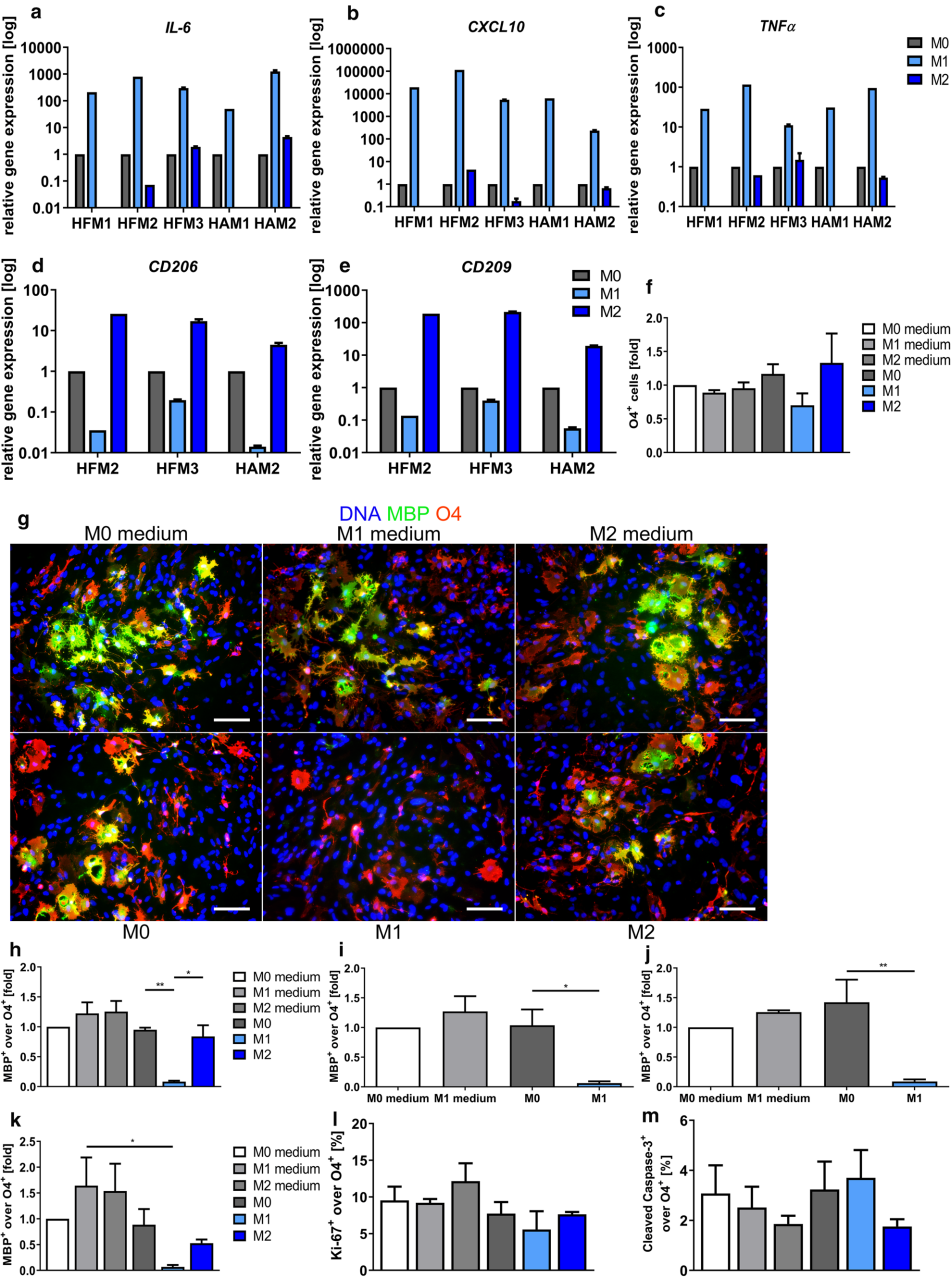
Supernatants of primary human M1, but not M2 or M0 polarized microglia inhibit the terminal differentiation of hiOL. Every experiment analyzing the effect of microglia supernatants on hiOL was performed with hiOL derived from three different donors. **a**–**e** To confirm successful polarization into M1 and M2 microglia, qRT-PCR was performed for *IL-6, CXCL10* and *TNFα* (M1) and *CD206* and *CD209* (M2). **f** Differentiation of hiOL in the presence of supernatants from M0, M1 or M2 polarized microglia from one fetal donor (HFM2) from day 4 to 21 resulted in no significant differences in the percentage of O4^+^ hiOL when comparing the effect of different supernatants with appropriate controls. **g**, **h** Culturing O4 sorted hiOL in the presence of supernatants from M1, but not M0 or M2 polarized microglia from one fetal donor (HFM3) from day 21 to 35 impaired significantly the differentiation of O4^+^ hiOL into MBP^+^ mature oligodendrocytes. **i**–**k** This was confirmed using supernatants from M1 and M0 polarized microglia from another fetal (HFM1) and one adult donor (HAM1) as well as supernatants from M0, M1 and M2 polarized microglia from a second adult donor (HAM2). **l** Analysis of percentages of actively dividing hiOL (Ki-67^+^ over O4^+^ cells) in the presence of supernatants from M0, M1 or M2 polarized microglia from one fetal donor (HFM2) from day 21 to 35 of differentiation did not reveal any significant differences. **m** Percentages of apoptotic hiOL (cleaved caspase 3^+^ over O4^+^ cells) in the presence of supernatants from M0, M1 or M2 polarized microglia from one fetal donor (HFM3) from day 21 to 35 of differentiation were not significantly altered. Scale bar in **g**: 100 µm. *hiOL* human iPSC-derived oligodendrocytes, *HFM* human fetal microglia, *HAM* human adult microglia, *IL-6* interleukin 6, *CXCL10* C-X-C motif chemokine 10, *TNFα* tumor necrosis factor alpha, *CD206/CD209* cluster of differentiation 206/209, *MBP* myelin basic protein

To further elucidate the impact of pro-inflammatory myeloid cells on oligodendroglial differentiation, we investigated the role of supernatants of activated microglia on hiOL [22]. Fetal and adult human primary microglia were stimulated for 48 h with either LPS and IFNγ to promote an M1 phenotype or with IL-4 and IL-13 to induce an M2 phenotype. In total, we obtained microglia supernatants from three fetal (HFM1-3) and two adult (HAM1, 2) donors. For validation of successful polarization, we determined the expression of typical M1 (*IL-6, CXCL10, TNFα*) and M2 cytokines (*CD206* and *CD209*) by qRT-PCR (Fig. 7a–e). Supernatants as well as the appropriate medium without the supernatants (controls) were added to differentiating hiOL from three different donors in a dilution of 1:10 from day 4 to 21 of differentiation to examine the effect on early differentiation. No differences in the percentage of O4^+^ cells were observed when comparing appropriate medium controls and supernatants (Fig. 7f) as assessed by flow cytometry for the oligodendroglial marker O4 which is expressed by immature and mature oligodendrocytes. To determine the effect of the supernatants on terminal differentiation, untreated O4^+^ hiOL sorted at day 21 of differentiation were exposed to M0, M1, and M2 microglia supernatants or medium controls for 14 days (dilution 1:10). ICC for MBP revealed significantly reduced numbers of mature MBP^+^ hiOL after application of M1 microglia supernatants but not M0 or M2 microglia supernatants from a fetal donor, while O4^+^ hiOL could still be detected (Fig. 7g, h). These results were validated using another fetal as well as two adult donors (Fig. 7i–k). To elucidate whether the reduced presence of MBP^+^ cells is caused by a decreased differentiation into mature hiOL or due to changes in cell death or proliferation, we analyzed the numbers of apoptotic and actively dividing hiOL by double ICC for cleaved caspase 3, respectively, Ki-67 and O4. Neither the percentage of apoptotic nor the percentage of actively dividing hiOL was significantly altered after application of microglia supernatants (Fig. 7l, m). Combined, these data demonstrate that supernatants from M1 polarized human microglia significantly inhibit the terminal differentiation of hiOL into mature MBP^+^ oligodendrocytes but do not affect apoptosis or proliferation of hiOL.

## Discussion

Promotion of remyelination represents a new and attractive treatment target in MS. In demyelinating animal models, the proliferation and differentiation of OPC are prerequisites for successful remyelination. In progressive disease stages, MS lesions display a relative preservation of OPC, but lack mature oligodendrocytes suggesting that a differentiation block contributes to remyelination failure in MS. This notion has been recently challenged by suggesting that not OPC, but mature oligodendrocytes are the major remyelinating cells in MS [34, 54, 71]. To further elucidate the mechanisms contributing to impaired remyelination in MS, we performed a detailed analysis of oligodendrocytes and remyelination in different MS lesion types. Our data suggest that different mechanisms contribute to impaired remyelination depending on lesion stage. In active/demyelinating lesions, remyelination occurs only in a subset of lesions despite the presence of high numbers of mature oligodendrocytes in all lesions indicating that an impaired formation of new myelin sheaths rather than a lack of mature oligodendrocytes contributes to insufficient remyelination in this lesion type. Furthermore, in mixed lesions, we observed an almost complete lack of remyelination and a relative increase of TMEM119^+^ microglia cells and iNOS^+^ pro-inflammatory myeloid cells. Supernatants of M1, but not M2 or M0 polarized microglia prevented terminal differentiation of hiOL indicating that pro-inflammatory microglia may contribute to impaired remyelination in mixed lesions.

The generally accepted view that OPC are prerequisite for successful remyelination is, among others, based on studies demonstrating that rodents in which proliferating oligodendrocytes were ablated by X-irradiation did not remyelinate [36]. Additionally, mature human oligodendrocytes transplanted into demyelinated and irradiated spinal cord lesions of rats did not contribute to remyelination [64]. One could speculate that X-irradiation may result in significant damage to the exposed tissue and other factors than the supposed incapability of mature oligodendrocytes may contribute to remyelination failure in these studies. However, in an elegant and more recent study, Crawford and colleagues found no evidence that pre-existing mature oligodendrocytes contribute to remyelination after toxin-induced demyelination using inducible reporter mouse lines and fate mapping [14]. Furthermore, a broad range of studies demonstrated that increased differentiation of OPC into mature myelinating oligodendrocytes results in accelerated remyelination in experimental animal models (for review see [25, 62]). Earlier histological studies using MS tissue samples demonstrated the presence of OPC and an almost complete absence of mature oligodendrocytes in mixed and inactive lesions [11, 39, 68, 70]. These findings together with the results from animal studies were interpreted as evidence for an impaired oligodendroglial differentiation as contributing factor for remyelination failure in MS. This view has been challenged by experimental studies reporting mature oligodendrocytes being connected to mature and remyelinated myelin sheaths in a cat as well as in a non-human primate model of de- and remyelination [19]. Additionally, the results of a recent study measuring the integration of ^14^C derived from nuclear testing into DNA of oligodendroglial lineage cells suggest that pre-existing oligodendrocytes and not proliferating OPC contribute to remyelination in MS [71]. Our data demonstrate the presence of remyelination and preservation of oligodendrocytes in highly inflammatory active/demyelinating lesions suggesting that oligodendrocytes are not the primary target of the immune attack in MS lesions. Furthermore, despite the presence of high numbers of mature oligodendrocytes, only a subset of lesions showed signs of remyelination. This indicates that not a differentiation block, but rather an impaired formation of new myelin sheaths contributes to remyelination failure in these lesions. Additionally, it raises the question which cells contributed to successful remyelination in active/demyelinating lesions with marked remyelination. We observed only a very low percentage of proliferating OPC in these lesions suggesting that these cells may not be major contributors to remyelination. Considering further the fact that OPC account for approximately 5% of the total oligodendroglial population and the relatively long time period required for differentiation of human OPC into mature oligodendrocytes [7, 12, 39], it appears unlikely that only recently differentiated OPC contributed to successful remyelination in active/demyelinating lesions, but instead favors a scenario in which also mature oligodendrocytes surviving the initial inflammatory attack contribute to remyelination. However, one has to keep in mind that Ki-67 only identifies cells which are actively dividing. The discrepancy between this scenario and experimental rodent studies might be explained by the fact that EAE, lysolecithine or cuprizone induced demyelination are characterized by a marked loss of mature oligodendrocytes [40, 49, 50] which is in contrast to our observations in active/demyelinating MS lesions. However, histology provides only a snapshot, and as long as no reliable markers for recently differentiated oligodendrocytes and remyelination are available, we will not be able to definitively clarify which cell population contributes to remyelination in MS. Importantly, our data also do not exclude the possibility that in other lesion types than active/demyelinating lesions, an oligodendroglial differentiation block contributes to impaired remyelination. Furthermore, remyelination is a complex process. It not only depends on the presence and absence of oligodendrocytes or its progenitors, but also requires axons susceptible for remyelination. Several studies for example have demonstrated that neuronal activity stimulates remyelination [3, 28, 55, 66]. Additionally, also astrocytes may influence the outcome of remyelination. They can be beneficial for remyelination by promotion of oligodendroglial differentiation and providing of cholesterol for new myelin sheaths, but have been also shown to impair remyelination by either activating remyelination inhibiting pathways such as the JAG1-NOTCH1 pathway or by modulating the extracellular matrix [10, 32, 35, 61, 72]. One also has to keep in mind that the biopsies were taken from patients with an untypical clinical presentation and, therefore, might not be representative for MS patients with a more typical disease onset. However, most of the patients diagnosed with an inflammatory demyelinating lesion consistent with MS, developed clinical definitive MS with a relapsing–remitting disease course [59].

The histological appearance of MS lesions changes over time. In acute and RRMS active lesions predominate; however, when MS progresses, the percentage of active lesions decreases, whereas the percentages of mixed and inactive lesions increase [26, 46]. Patients with a more severe disease course have a higher percentage of mixed lesions suggesting that mixed lesions contribute significantly to disease progression [46]. Interestingly, we observed an almost complete absence of remyelination in mixed lesions in contrast to active or inactive lesions. This is in line with an earlier publication by Luchetti and colleagues who reported an inverse correlation between mixed versus remyelinated lesions [46]. Remyelination is frequently occurring at the lesion border; however, mixed lesions are characterized by a rim of myeloid cells at the lesion border suggesting that tissue environment in the rim of mixed lesions prevents successful remyelination. Interestingly, we observed a relative increase in TMEM119^+^ and iNOS^+^ and a decrease of CD163^+^ myeloid cells in the rim of mixed lesions compared to active lesions; however, one has also to keep in mind that TMEM119 is a homeostatic microglia marker whose expression decreases with activation [4]. In EAE, myeloid cells change from an iNOS^+^ pro-inflammatory and tissue-destructive phenotype to an iNOS^−^ tissue repair phenotype and this may be mediated by other immune cells, such as T and B cells [23, 43, 58]. This is in contrast to mixed MS lesions where at least a subset of myeloid cells preserves an iNOS^+^ phenotype. To further understand consequences of pro-inflammatory myeloid cells for oligodendrocytes, we exposed hiOL to the supernatants of pro- and anti-inflammatory microglia. We observed a marked decrease in the terminal differentiation into MBP^+^ oligodendrocytes after exposure to supernatants from pro-inflammatory human microglia, whereas supernatants from microglia polarized into an M0 or an M2 phenotype did not affect oligodendroglial differentiation. This is contrary to results from rodent studies in which supernatants from M0 and M1 polarized microglia had no effect on oligodendroglial differentiation, whereas the supernatants from M2 polarized microglia promoted oligodendroglial differentiation [52]. Microvesicles isolated from either pro- or anti-inflammatory rat microglia both promoted the differentiation of rat oligodendrocytes [44]. These different results suggest that there might be significant differences between rodent and human oligodendrocytes with respect to the reaction to an inflammatory milieu. However, also the protocols for the polarization into pro- and anti-inflammatory microglial cells were slightly different and this might have also affected the outcome. Furthermore, other immune cells, such as T and B cells, have been reported to influence remyelination, either via direct effects on oligodendrocytes or by modulating myeloid cells [17].

We observed a comparable percentage of lesions with marked remyelination in active and inactive lesions. Therefore, it might be tempting to speculate that active lesions with marked remyelination become remyelinated inactive lesions, whereas active lesions lacking marked remyelination turn into mixed lesions and potentially afterwards into inactive lesions lacking remyelination. However, as mentioned above, histological studies provide only a snapshot and imaging studies demonstrated repeated waves of de- and remyelination in MS lesions [6]. Imaging markers for the different lesions types and longitudinal imaging studies are required to understand how lesions develop over time and how they contribute to disease progression.

In summary, our data suggest that the underlying causes for remyelination failure in MS are lesion stage dependent. Whereas in active/demyelinating lesions, lack of myelin sheath formation contributes to remyelination failure, in inactive and mixed lesions oligodendroglial loss and a hostile tissue environment may prevent successful remyelination. Since pharmacological approaches that focus exclusively on promotion of oligodendroglial differentiation to enhance remyelination might fail, treatment strategies targeting multiple of the steps required for successful remyelination, namely oligodendroglial proliferation, migration, differentiation, myelin sheath formation, and survival should be considered. Our data also suggest that promotion of remyelination in progressive MS in which mixed and inactive lesions dominate may require different treatment approaches compared to RRMS characterized by more active lesions. To understand the molecular mechanisms driving and preventing remyelination and to develop new treatment approaches which successfully promote remyelination in MS, better animal models are required which mimic the pathological hallmarks of the different lesion types.

## Electronic supplementary material

Below is the link to the electronic supplementary material.

Supplementary file1 (PDF 245 kb)

## References

[1] Adelman G, Rane SG, Villa KF (2013). The cost burden of multiple sclerosis in the United States: a systematic review of the literature. J Med Econ.

[2] Albert M, Antel J, Bruck W, Stadelmann C (2007). Extensive cortical remyelination in patients with chronic multiple sclerosis. Brain Pathol.

[3] Barres BA, Raff MC (1993). Proliferation of oligodendrocyte precursor cells depends on electrical activity in axons. Nature.

[4] Bennett ML, Bennett FC, Liddelow SA, Ajami B, Zamanian JL, Fernhoff NB (2016). New tools for studying microglia in the mouse and human CNS. Proc Natl Acad Sci USA.

[5] Bodini B, Veronese M, Garcia-Lorenzo D, Battaglini M, Poirion E, Chardain A (2016). Dynamic imaging of individual remyelination profiles in multiple sclerosis. Ann Neurol.

[6] Brown RA, Narayanan S, Arnold DL (2014). Imaging of repeated episodes of demyelination and remyelination in multiple sclerosis. Neuroimage Clin.

[7] Butt A, Kiff J, Hubbard P, Berry M (2002). Synantocytes: new functions for novel NG2 expressing glia. J Neurocytol.

[8] Cadavid D, Balcer L, Galetta S, Aktas O, Ziemssen T, Vanopdenbosch L (2017). Safety and efficacy of opicinumab in acute optic neuritis (RENEW): a randomised, placebo-controlled, phase 2 trial. Lancet Neurol.

[9] Cadavid D, Mellion M, Hupperts R, Edwards KR, Calabresi PA, Drulovic J (2019). Safety and efficacy of opicinumab in patients with relapsing multiple sclerosis (SYNERGY): a randomised, placebo-controlled, phase 2 trial. Lancet Neurol.

[10] Cantuti-Castelvetri L, Fitzner D, Bosch-Queralt M, Weil MT, Su M, Sen P (2018). Defective cholesterol clearance limits remyelination in the aged central nervous system. Science.

[11] Chang A, Tourtellotte WW, Rudick R, Trapp BD (2002). Premyelinating oligodendrocytes in chronic lesions of multiple sclerosis. N Engl J Med.

[12] Chanoumidou K, Mozafari S, Baron-Van Evercooren A, Kuhlmann T (2020). Stem cell derived oligodendrocytes to study myelin diseases. Glia.

[13] Chen JT, Collins DL, Atkins HL, Freedman MS, Arnold DL (2008). Magnetization transfer ratio evolution with demyelination and remyelination in multiple sclerosis lesions. Ann Neurol.

[14] Crawford AH, Tripathi RB, Foerster S, McKenzie I, Kougioumtzidou E, Grist M (2016). Pre-existing mature oligodendrocytes do not contribute to remyelination following toxin-induced spinal cord demyelination. Am J Pathol.

[15] Dendrou CA, Fugger L, Friese MA (2015). Immunopathology of multiple sclerosis. Nat Rev Immunol.

[16] Deshmukh VA, Tardif V, Lyssiotis CA, Green CC, Kerman B, Kim HJ (2013). A regenerative approach to the treatment of multiple sclerosis. Nature.

[17] Dombrowski Y, O'Hagan T, Dittmer M, Penalva R, Mayoral SR, Bankhead P (2017). Regulatory T cells promote myelin regeneration in the central nervous system. Nat Neurosci.

[18] Duncan ID, Brower A, Kondo Y, Curlee JF, Schultz RD (2009). Extensive remyelination of the CNS leads to functional recovery. Proc Natl Acad Sci USA.

[19] Duncan ID, Radcliff AB, Heidari M, Kidd G, August BK, Wierenga LA (2018). The adult oligodendrocyte can participate in remyelination. Proc Natl Acad Sci USA.

[20] Durafourt BA, Moore CS, Blain M, Antel JP (2013). Isolating, culturing, and polarizing primary human adult and fetal microglia. Methods Mol Biol.

[21] Durafourt BA, Moore CS, Zammit DA, Johnson TA, Zaguia F, Guiot MC (2012). Comparison of polarization properties of human adult microglia and blood-derived macrophages. Glia.

[22] Ehrlich M, Mozafari S, Glatza M, Starost L, Velychko S, Hallmann AL (2017). Rapid and efficient generation of oligodendrocytes from human induced pluripotent stem cells using transcription factors. Proc Natl Acad Sci USA.

[23] El Behi M, Sanson C, Bachelin C, Guillot-Noel L, Fransson J, Stankoff B (2017). Adaptive human immunity drives remyelination in a mouse model of demyelination. Brain.

[24] Evans FL, Dittmer M, de la Fuente AG, Fitzgerald DC (2019). Protective and regenerative roles of T cells in central nervous system disorders. Front Immunol.

[25] Franklin RJM, Ffrench-Constant C (2017). Regenerating CNS myelin—from mechanisms to experimental medicines. Nat Rev Neurosci.

[26] Frischer JM, Weigand SD, Guo Y, Kale N, Parisi JE, Pirko I (2015). Clinical and pathological insights into the dynamic nature of the white matter multiple sclerosis plaque. Ann Neurol.

[27] Galloway DA, Gowing E, Setayeshgar S, Kothary R (2020). Inhibitory milieu at the multiple sclerosis lesion site and the challenges for remyelination. Glia.

[28] Gibson EM, Purger D, Mount CW, Goldstein AK, Lin GL, Wood LS (2014). Neuronal activity promotes oligodendrogenesis and adaptive myelination in the mammalian brain. Science.

[29] Goldschmidt T, Antel J, Konig FB, Brück W, Kuhlmann T (2009). Remyelination capacity of the MS brain decreases with disease chronicity. Neurology.

[30] Green AJ, Gelfand JM, Cree BA, Bevan C, Boscardin WJ, Mei F (2017). Clemastine fumarate as a remyelinating therapy for multiple sclerosis (ReBUILD): a randomised, controlled, double-blind, crossover trial. Lancet.

[31] Hoftberger R, Fink S, Aboul-Enein F, Botond G, Olah J, Berki T (2010). Tubulin polymerization promoting protein (TPPP/p25) as a marker for oligodendroglial changes in multiple sclerosis. Glia.

[32] Houben E, Janssens K, Hermans D, Vandooren J, Van den Haute C, Schepers M (2020). Oncostatin M-induced astrocytic tissue inhibitor of metalloproteinases-1 drives remyelination. Proc Natl Acad Sci USA.

[33] Irvine KA, Blakemore WF (2008). Remyelination protects axons from demyelination-associated axon degeneration. Brain.

[34] Jakel S, Agirre E, Mendanha Falcao A, van Bruggen D, Lee KW, Knuesel I (2019). Altered human oligodendrocyte heterogeneity in multiple sclerosis. Nature.

[35] John GR, Shankar SL, Shafit-Zagardo B, Massimi A, Lee SC, Raine CS (2002). Multiple sclerosis: re-expression of a developmental pathway that restricts oligodendrocyte maturation. Nat Med.

[36] Keirstead HS, Blakemore WF (1997). Identification of post-mitotic oligodendrocytes incapable of remyelination within the demyelinated adult spinal cord. J Neuropathol Exp Neurol.

[37] Kuhlmann T, Lingfeld G, Bitsch A, Schuchardt J, Brück W (2002). Acute axonal damage in multiple sclerosis is most extensive in early disease stages and decreases over time. Brain.

[38] Kuhlmann T, Ludwin S, Prat A, Antel J, Bruck W, Lassmann H (2017). An updated histological classification system for multiple sclerosis lesions. Acta Neuropathol.

[39] Kuhlmann T, Miron V, Cui Q, Wegner C, Antel J, Brück W (2008). Differentiation block of oligodendroglial progenitor cells as a cause for remyelination failure in chronic multiple sclerosis. Brain.

[40] Kuhlmann T, Remington L, Maruschak B, Owens T, Bruck W (2007). Nogo-A is a reliable oligodendroglial marker in adult human and mouse CNS and in demyelinated lesions. J Neuropathol Exp Neurol.

[41] Lambert C, Ase AR, Seguela P, Antel JP (2010). Distinct migratory and cytokine responses of human microglia and macrophages to ATP. Brain Behav Immun.

[42] Lloyd AF, Miron VE (2019). The pro-remyelination properties of microglia in the central nervous system. Nat Rev Neurol.

[43] Locatelli G, Theodorou D, Kendirli A, Jordao MJC, Staszewski O, Phulphagar K (2018). Mononuclear phagocytes locally specify and adapt their phenotype in a multiple sclerosis model. Nat Neurosci.

[44] Lombardi M, Parolisi R, Scaroni F, Bonfanti E, Gualerzi A, Gabrielli M (2019). Detrimental and protective action of microglial extracellular vesicles on myelin lesions: astrocyte involvement in remyelination failure. Acta Neuropathol.

[45] Lucchinetti C, Brück W, Parisi J, Scheithauer B, Rodriguez M, Lassmann H (2000). Heterogeneity of multiple sclerosis lesions: implications for the pathogenesis of demyelination. Ann Neurol.

[46] Luchetti S, Fransen NL, van Eden CG, Ramaglia V, Mason M, Huitinga I (2018). Progressive multiple sclerosis patients show substantial lesion activity that correlates with clinical disease severity and sex: a retrospective autopsy cohort analysis. Acta Neuropathol.

[47] Manrique-Hoyos N, Jurgens T, Gronborg M, Kreutzfeldt M, Schedensack M, Kuhlmann T (2012). Late motor decline after accomplished remyelination: impact for progressive multiple sclerosis. Ann Neurol.

[48] Marques S, Zeisel A, Codeluppi S, van Bruggen D, Mendanha Falcao A, Xiao L (2016). Oligodendrocyte heterogeneity in the mouse juvenile and adult central nervous system. Science.

[49] Matsushima GK, Morell P (2001). The neurotoxicant, cuprizone, as a model to study demyelination and remyelination in the central nervous system. Brain Pathol.

[50] Mei F, Fancy SP, Shen YA, Niu J, Zhao C, Presley B (2014). Micropillar arrays as a high-throughput screening platform for therapeutics in multiple sclerosis. Nat Med.

[51] Mi S, Miller RH, Tang W, Lee X, Hu B, Wu W (2009). Promotion of central nervous system remyelination by induced differentiation of oligodendrocyte precursor cells. Ann Neurol.

[52] Miron VE, Boyd A, Zhao JW, Yuen TJ, Ruckh JM, Shadrach JL (2013). M2 microglia and macrophages drive oligodendrocyte differentiation during CNS remyelination. Nat Neurosci.

[53] Najm FJ, Madhavan M, Zaremba A, Shick E, Karl RT, Factor DC (2015). Drug-based modulation of endogenous stem cells promotes functional remyelination in vivo. Nature.

[54] Nave KA, Ehrenreich H (2019). Time to revisit oligodendrocytes in multiple sclerosis. Nat Med.

[55] Ortiz FC, Habermacher C, Graciarena M, Houry PY, Nishiyama A, Nait Oumesmar B (2019). Neuronal activity in vivo enhances functional myelin repair. JCI Insight.

[56] Patani R, Balaratnam M, Vora A, Reynolds R (2007). Remyelination can be extensive in multiple sclerosis despite a long disease course. Neuropathol Appl Neurobiol.

[57] Patrikios P, Stadelmann C, Kutzelnigg A, Rauschka H, Schmidtbauer M, Laursen H (2006). Remyelination is extensive in a subset of multiple sclerosis patients. Brain.

[58] Pennati A, Nylen EA, Duncan ID, Galipeau J (2020). Regulatory B cells normalize CNS myeloid cell content in a mouse model of multiple sclerosis and promote oligodendrogenesis and remyelination. J Neurosci.

[59] Pittock SJ, McClelland RL, Achenbach SJ, Konig F, Bitsch A, Bruck W (2005). Clinical course, pathological correlations, and outcome of biopsy proved inflammatory demyelinating disease. J Neurol Neurosurg Psychiatry.

[60] Reinhardt P, Glatza M, Hemmer K, Tsytsyura Y, Thiel CS, Hoing S (2013). Derivation and expansion using only small molecules of human neural progenitors for neurodegenerative disease modeling. PLoS ONE.

[61] Segel M, Neumann B, Hill MFE, Weber IP, Viscomi C, Zhao C (2019). Niche stiffness underlies the ageing of central nervous system progenitor cells. Nature.

[62] Stangel M, Kuhlmann T, Matthews PM, Kilpatrick TJ (2017). Achievements and obstacles of remyelinating therapies in multiple sclerosis. Nat Rev Neurol.

[63] Tallantyre EC, Bo L, Al-Rawashdeh O, Owens T, Polman CH, Lowe JS (2010). Clinico-pathological evidence that axonal loss underlies disability in progressive multiple sclerosis. Mult Scler.

[64] Targett M, Sussman J, Scolding N, O'Leary MT, Compston D, Blakemore WF (1996). Failure to achieve remyelination of demyelinated rat axons following transplantation of glial cells obtained from the adult human brain. Neuropathol Appl Neurobiol.

[65] Trapp BD, Peterson J, Ransohoff RM, Rudick R, Mork S, Bo L (1998). Axonal transection in the lesions of multiple sclerosis. N Engl J Med.

[66] Wake H, Ortiz FC, Woo DH, Lee PR, Angulo MC, Fields RD (2015). Nonsynaptic junctions on myelinating glia promote preferential myelination of electrically active axons. Nat Commun.

[67] Weider M, Starost LJ, Groll K, Kuspert M, Sock E, Wedel M (2018). Nfat/calcineurin signaling promotes oligodendrocyte differentiation and myelination by transcription factor network tuning. Nat Commun.

[68] Wolswijk G (1998). Chronic stage multiple sclerosis lesions contain a relatively quiescent population of oligodendrocyte precursor cells. J Neurosci.

[69] Wolswijk G (2002). Oligodendrocyte precursor cells in the demyelinated spinal cord. Brain.

[70] Wolswijk G (1998). Oligodendrocyte regeneration in the adult rodent CNS and the failure of this process in multiple sclerosis. Prog Brain Res.

[71] Yeung M, Djelloul M, Steiner E, Bernard S, Salehpour M, Possnert G (2019). Dynamics of oligodendrocyte generation in multiple sclerosis. Nature.

[72] Zhang Y, Argaw AT, Gurfein BT, Zameer A, Snyder BJ, Ge C (2009). Notch1 signaling plays a role in regulating precursor differentiation during CNS remyelination. Proc Natl Acad Sci USA.

