# Supplementary material for: Lesion stage-dependent causes for impaired remyelination in MS
Source: Acta Neuropathol. 2020 Jul 24;140(3):359–75. doi: 10.1007/s00401-020-02189-9 (PMC7424408; doi:10.1007/s00401-020-02189-9)
Supplement: Supplementary file 1 — Supplementary file1 (PDF 245 kb) [file 401_2020_2189_MOESM1_ESM.pdf]

Lesion stage dependent causes for impaired remyelination in MS

Acta Neuropathologica

Katharina Heß[1], Laura Starost[1,2], Nicholas W. Kieran[3], Christian Thomas[1], Jack Antel[3], Gianvito Martino[4], Inge Huitinga[5], Luke Healy[3], Tanja Kuhlmann[1]

[1]Institut für Neuropathologie, Universitätsklinikum Münster, 48149 Münster, Germany; [2]Max Planck Institut für molekulare Biomedizin, 48149 Münster, Germany; [3]Montreal Neurological Institute, McGill University, Montreal, Canada; [4]Neuroimmunology Unit, Institute of Experimental Neurology, Division of Neuroscience, IRCCS San Raffaele Hospital, 20132 Milan, Italy; [5]Department of Neuroimmunology, The Netherlands Institute for Neuroscience, Amsterdam, The Netherlands.

Corresponding author:

Tanja Kuhlmann, Institute of Neuropathology, University Hospital Münster, Pottkamp 2, 48149 Münster, Germany

Telephone +49 251 83 52614, Fax +49 251 83 56971

Email: tanja.kuhlmann@ukmuenster.de

SUPPLEMENTARY TABLES AND FIGURES

Table S1 Source and dilution of antibodies

Antibody	Origin	Dilution	Source	Application
CD3	Mouse	1:25	M7254, Dako	IHC
CD20	Mouse	1:700	M0755, Dako	IHC
CD68	Mouse	1:200	M0814, Dako	IHC
CD163	Mouse	1:200	NCL-L-CD163, Leica/Novocastra	IHC
CD206	Rabbit	1:100	HPA004114, Sigma	IHC
Cleaved Caspase 3	Rabbit	1:300	MAB835, R&D	ICC
CNPase	Mouse	1:1000	836404, BioLegend	IHC
iNOS	Rabbit	1:1000	ABN26, Millipore	IHC
Ki-67	Rabbit	1:200	Ab16667, Abcam	IHC
Ki-67	Rabbit	1:250	Ab16667, Abcam	ICC
MBP	Rat	1:200	Ab7349, Abcam	IHC
MBP	Rat	1:50	Ab7349, Abcam	ICC
NOGOA	Rabbit	1:200	AB5664P, Millipore	IHC
O4	Mouse	1:1000	MAB1326, R&D	ICC
OLIG2	Mouse	1:200	387M-16, Medac	IHC
TMEM119	Rabbit	1:500	HPA051870, Sigma	IHC
TPPP/p25	Rabbit	1:500	Ab92305, Abcam	IHC

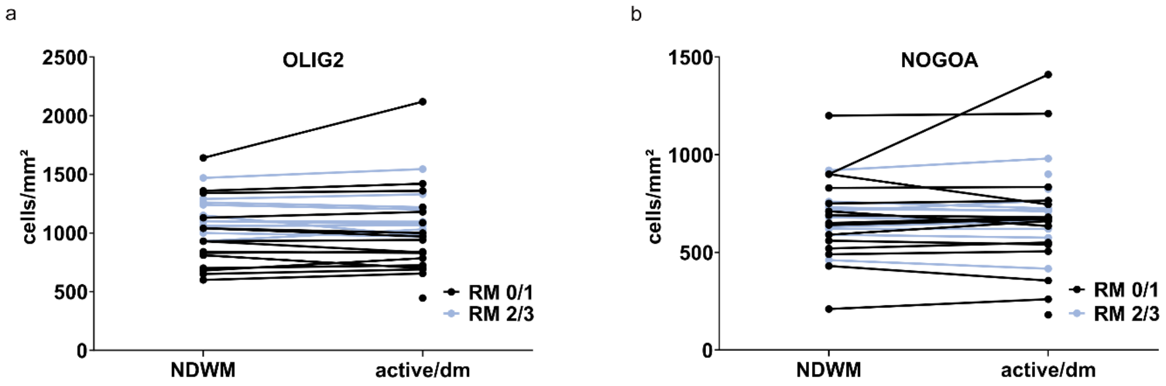

Fig. S1 Preservation of oligodendrocytes in active/demyelinating lesions. **a and b** When comparing tissue samples containing NDWM and active/demyelinating lesions no significant differences in numbers of OLIG2$^+$ and NOGOA$^+$ oligodendrocytes were observed. Lesions with marked remyelination are indicated in blue, lesions with limited remyelination in black. OLIG2 = Oligodendrocyte Transcription Factor 2; NDWM = non-demyelinated white matter; active/dm = active/demyelinating lesions; RM 0/1 = remyelination score 0 or 1; RM 2/3 = remyelination score 2 or 3

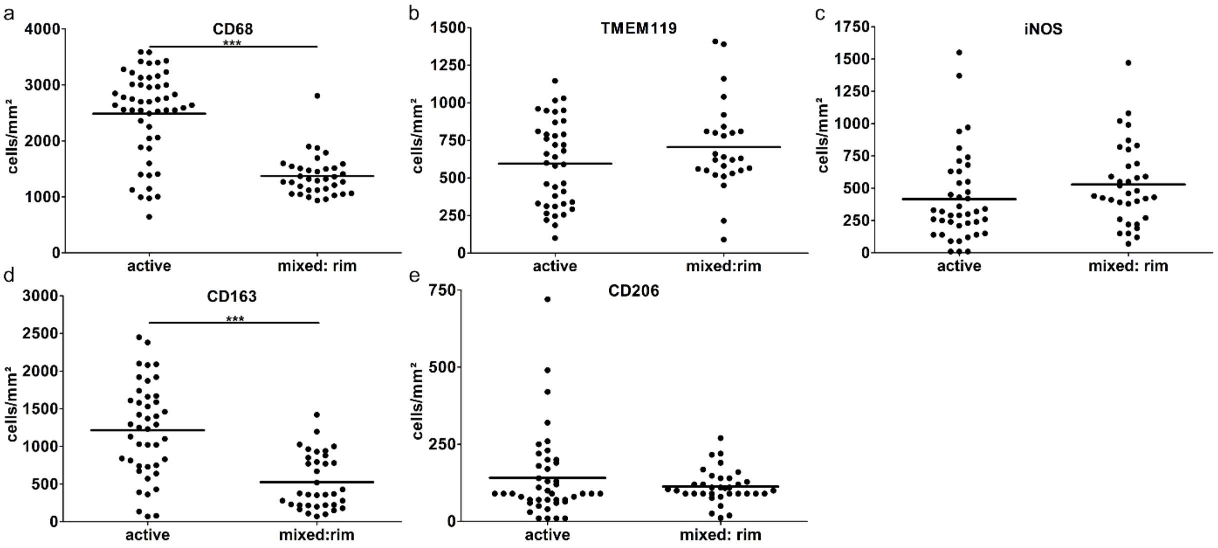

Fig. S2 Absolute numbers of myeloid cells in active and mixed lesions. **a to e** Quantification of absolute numbers of CD68$^+$, TMEM119$^+$, iNOS$^+$, CD163$^+$ and CD206$^+$ myeloid cells in active lesion and in the rim of mixed lesions. CD68/163/206 = Cluster of Differentiation 68/163/206; TMEM119 = Transmembrane Protein 119; iNOS = inducible Nitric Oxide Synthase; active = active lesions; mixed: rim = rim of mixed active/inactive lesions
